# Functional and Clinical Characterization of Tumor-Infiltrating T Cell Subpopulations in Hepatocellular Carcinoma

**DOI:** 10.3389/fgene.2020.586415

**Published:** 2020-09-30

**Authors:** Jianguo Li, Jin Zhou, Shuangshuang Kai, Can Wang, Daijun Wang, Jiying Jiang

**Affiliations:** Schools of Medicine and Pharmacy, Weifang Medical University, Weifang, China

**Keywords:** T cell infiltrating level, driver gene, cell cycle progression, immune checkpoint regulator, immunotherapeutic target

## Abstract

Tumor-infiltrating T-lymphocytes are defined as T-lymphocytes that infiltrated into tumor tissues; however, their composition, clinical significance, and underlying mechanism in hepatocellular carcinoma (HCC) and adjacent non-tumor tissues are still not completely understood. Herein, we collected marker genes of T cell subpopulations from a previous study and estimated their relative infiltrating levels in HCC and adjacent non-tumor tissues. Specifically, the infiltrating levels of all the T cells were significantly reduced in HCC as compared with non-tumor tissues. Unsupervised clustering of the HCC samples by the T cell infiltrating levels revealed that the HCC samples could be clearly classified into two groups. The driver genes, including *PTK2B*, *ATM*, *PIK3C2B*, and *KIT*, and several CNAs were observed to be associated with reduced T cell infiltrating levels. Particularly, deletion of TP53 more frequently occurred in low T cell infiltration HCC samples and resulted in its downregulation and cell cycle progression, indicating that cell cycle progression was closely associated with reduced T cell infiltration. In contrast, for the samples with high infiltration T cells, its immune evasion might be regulated by the immune checkpoint regulators, such as PD-1/PD-L1 and CTLA4. Moreover, Olaparib, one of the PARP inhibitors, and immune checkpoint inhibitors might be therapeutic candidates for the samples from the two T cell infiltrating clusters. Clinically, the tumor-infiltrating levels of cytotoxic CD4 cell, Mucosal associated invariant T (MAIT) cell, and exhausted CD8^+^ T cell might be used as predictors for vascular invasion, recurrence, and overall survival. Collectively, we systematically evaluated the clinical significance and potential molecular mechanisms of tumor-infiltrating T cell subpopulations in hepatocellular carcinoma, which might broaden our insights into the immunological features of HCC and provide potential immunotherapeutic targets.

## Introduction

Hepatocellular carcinoma (HCC) is the most prevalent primary liver cancer worldwide, with an increasing annual incidence (Pinyol and Llovet, 2014; Llovet et al., 2018). Etiologic factors of HCC mainly include hepatitis B/C viruses, alcohol abuse, and cirrhosis (Grandhi et al., 2016). Cirrhosis is an important indicator clinically, and patients are facing a higher risk of developing HCC and sometimes their lives are threatened (Mazzanti et al., 2008; Hartke et al., 2017). Poor prognoses of HCC are related to the fact that many patients have advanced-stage HCC upon diagnosis, where chemotherapy, radiotherapy, surgical resection, and transplantation are no longer curative (Fu et al., 2019). However, immunotherapy trials have hinted that immune checkpoint inhibition could be of better help to HCC patients when compared with other known therapies (Harding et al., 2016).

Since many HCC cases are associated with chronic inflammation, the assessment of inflammatory tumor microenvironment and its components seems promising (Chew et al., 2010; Hendry et al., 2017). Tumor infiltrating T-lymphocytes and their density are known to exhibit prognostic values in various types of solid tumors, and they are associated with the expression of certain biomarkers, which could serve as therapeutic targets (Ren and Zhang, 2019). One example is that for HBV-related HCC cases, the outcome of HDV/HBV infection could be related to virus-specific CD8 + T cells, and there is an established immunoscore based on immune infiltration to predict their prognosis (Deterding et al., 2009; Chen et al., 2019). To our knowledge, HCC often exhibits distinctive intratumoral immune states, and it is reported that a combination of low intratumoral regulatory T cells (Tregs) and high intratumoral activated CD8 + cytotoxic cells (CTLs) can serve as prognostic factors of HCC, as Tregs are associated with HCC invasiveness and CTLs could help mediate anti-tumor immune response (Gao et al., 2007; Thorsson et al., 2018). However, none of CD3^+^, CD4^+^, CD8^+^ TILs had independent prognostic value (Gao et al., 2007), suggesting that a further illustration of HCC-infiltrating T cells and identification of biomarkers in HCC immune responses are needed. The mucosal-associated invariant T (MAIT) cells are enriched in liver; however, its immunomodulatory role in HCC is controversial. Recently, HCC-infiltrating MAIT cells were found to be impaired and even reprogrammed in HCC (Duan et al., 2019), and their functionality had been altered from anticancer activity to tumor-promoting activity. In the present study, we aimed to explore the tumor-infiltrating levels of T cell subpopulations in HCC and non-tumor tissues, and anticipated we would evaluate their clinical significance and potential molecular mechanism in HCC.

## Materials and Methods

### The Gene Expression, Somatic Mutation, DNA Methylation, and Copy Number Alteration Data

The level-3 data of gene expression profiles, somatic mutations, DNA methylation, and copy number alterations from TCGA LIHC cohort (Cancer Genome Atlas Research Network, 2017) were downloaded from TCGA Data Portal. The T cell subpopulations and the corresponding marker genes were obtained from previous study (Zheng et al., 2017). The normalized gene expression data of HCC samples with vascular invasion and recurrent tissues were downloaded from Gene Expression Omnibus (GEO) database with accession number GSE20017 (Minguez et al., 2011) (*n* = 135) and GSE56545 (*n* = 42), respectively. Moreover, the independent gene expression dataset with accession SRP068976 (*n* = 100) (Liu et al., 2016) was obtained in Sequence Read Archive (SRA) database.

### The Tumor-Infiltrating Levels of T Cell Subpopulations

Following the previous study (Senbabaoglu et al., 2016), we estimated the tumor-infiltrating levels of T cell subpopulations using single-sample gene set enrichment analysis (ssGSEA), which used gene expression profiles and marker genes of T cells as input and returned the relative infiltrating levels of each T cell type for each sample. The ssGSEA was implemented in R/Bioconductor package GSVA (Hanzelmann et al., 2013), which could estimate the relative activity of immune cells.

### Unsupervised Clustering Analysis

The K-means clustering analysis was employed to cluster the HCC samples based on the T cell relative infiltrating levels. The optimal number of clusters was determined by 30 indices and implemented in R package NbClust (Charrad et al., 2014), which classified the HCC samples into high and low immune infiltrating clusters.

### The Gene Set Enrichment Analysis (GSEA)

The cell cycle related genes were obtained from Kyoto Encyclopedia of Genes and Genomes (KEGG) database (Kanehisa and Goto, 2000), and the genes were pre-ranked by the *t*-statistics that represent the difference between the High and Low groups. The GSEA (Subramanian et al., 2005) and its visualization were implemented in R fgsea package (Korotkevich et al., 2019).

### The Correlation Analysis

The Pearson correlation analysis was conducted to evaluate the correlation between two objects. The correlation coefficients and *P*-values for the correlation tests were calculated by R *cor* and *cor.test* function, respectively.

### The Survival Analysis

The Cox proportional hazard regression analysis was used in this study. The high and low infiltrating levels of the T cells were determined by scanning the threshold between 25 and 75% with the optimal statistical significance by Log-rank tests.

### Statistical Analyses

The Wilcoxon rank sum test and *t*-test were used to test the two-sample mean differences. The proportion test or Chi-square test was used to test the two-sample differences of proportions. All these analyses were implemented in R with version 3.6.0. *P* < 0.05 was deemed as statistically significant.

## Results

### The Tumor-Infiltrating Levels of T Cell Subpopulations in Hepatocellular Carcinoma and Non-tumor Tissues

To explore the tumor-infiltrating levels of T cell subpopulations in hepatocellular carcinoma and non-tumor tissues, we first collected the marker genes of 11 T cell types identified by previous study (Zheng et al., 2017) and excluded two of these cell types due to lack of enough marker genes (*n* > 30). Specifically, a total of 961 marker genes representing nine T cell types including naive CD8^+^ T cell, exhausted CD4^+^ T cell, cytotoxic CD4 cell, effector memory CD8^+^ T cell, Mucosal associated invariant T (MAIT) cell, exhausted CD8^+^ T cell, naive CD4^+^ T cell, peripheral T regulatory cell (Treg.), and tumor Treg. were used for the downstream data analysis.

**FIGURE 1:**
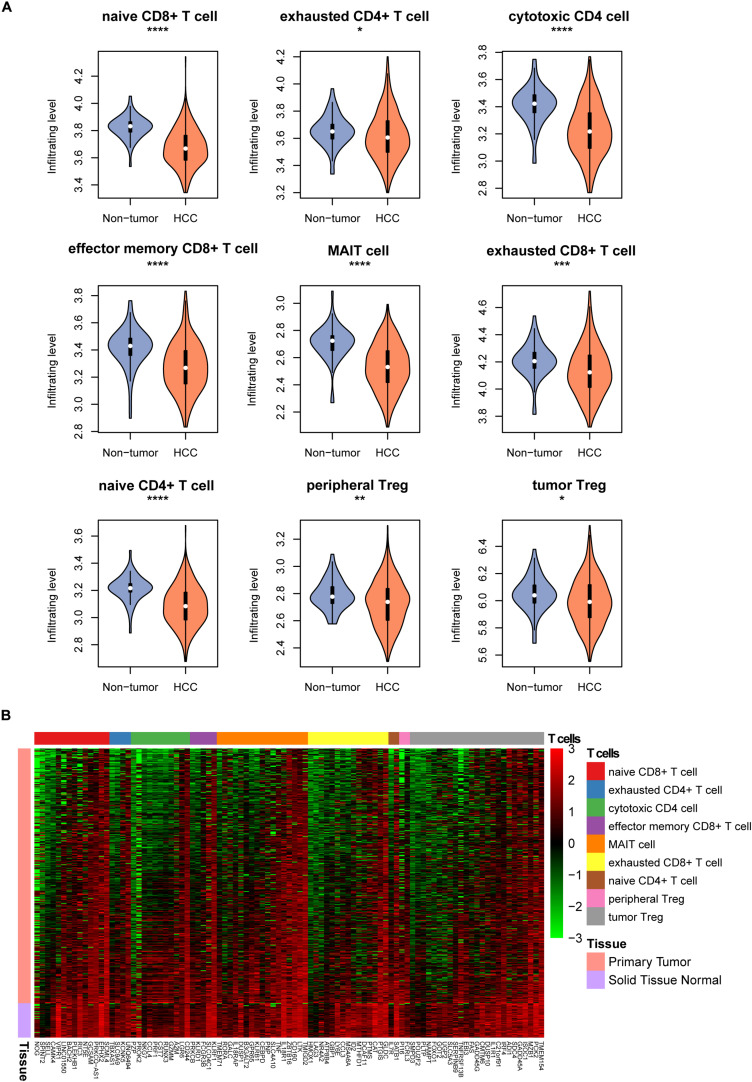
The tumor-infiltrating patterns of T cell subpopulations in HCC. **(A)** The T cell infiltrating levels in HCC and non-tumor tissues. The differential infiltrating levels were evaluated by Wilcoxon rank sum test. **(B)** The expression profiles of the marker genes of T cells in HCC and non-tumor tissues. *p < 0.05; **p < 0.01; ***p < 0.001; ****p < 0.0001.

To estimate the tumor-infiltrating levels of T cell subpopulations in hepatocellular carcinoma and non-tumor tissues, we conducted single-sample gene set enrichment analysis (ssGSEA) of the gene expression profiles of 371 hepatocellular carcinoma (HCC) and 50 non-tumor samples from TCGA liver hepatocellular carcinoma (LIHC) cohort (Supplementary Table S1). For the nine T cell types, their relative tumor-infiltrating levels were reduced in the HCC tissues as compared to the non-tumor tissues (Figure 1A, Wilcoxon rank sum test, *P* < 0.05). Particularly, the naive CD8^+^ T cell, cytotoxic CD4 cell, effector memory CD8^+^ T cell, MAIT cell, and naive CD4^+^ T cell had the highest statistical significances (*P* < 0.0001). Moreover, the tumor-infiltrating levels of the T cell types except exhausted CD4 + T cell and tumor Treg. were also observed to be reduced in HCC in an independent dataset (Liu et al., 2016) (SRA accession number SRP068976, Supplementary Figure S1). Consistently, the marker genes of these T cells were also downregulated in HCC tissues (Figure 1B). These findings indicated that the tumor-infiltrating levels of T cell subpopulations were reduced in hepatocellular carcinoma.

### The T Cell-Related HCC Subgroups and Subgroup-Specific Genomic Alterations

**FIGURE 2:**
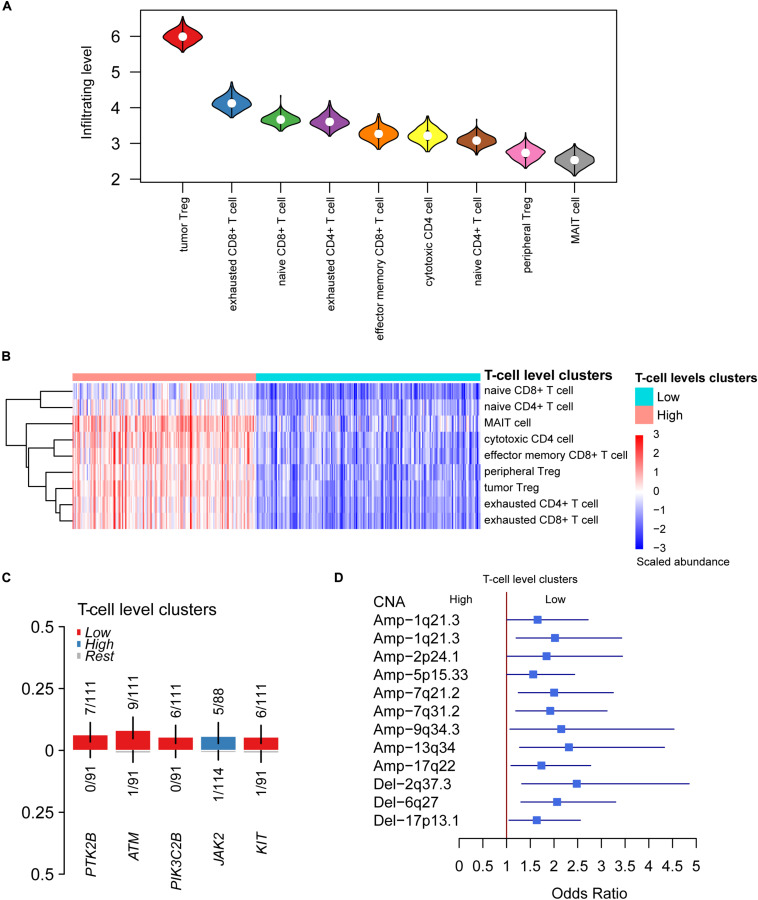
The differential infiltrating levels of T cells between the two HCC T-cell level clusters. **(A)** The HCC-infiltrating levels between T cell subpopulations. The cells were ordered by the median of the relative infiltrating levels. **(B)** The unsupervised clustering-identified T cell level clusters. The red and green color bands on the top represent the High and Low T cell level clusters. **(C,D)** The somatic mutations and copy number alterations (CNAs) preferentially mutated in the two T cell level clusters.

With the relative tumor-infiltrating levels of T cells, we observed that tumor Treg. and exhausted CD8^+^ T cells had significantly higher infiltrating levels in HCC than other cell types (Figure 2A), suggesting that the two cell types might play a key role in immune evasion of HCC. Furthermore, the K-means clustering analysis was conducted on the tumor-infiltrating levels of T cells. The optimal number of clusters was determined by 30 indices and implemented in R package NbClust. As shown in Figure 2B, the HCC samples were stratified into two groups, termed as High and Low clusters.

To characterize the genomic alterations associated with T cell infiltrating levels, we compared the somatic mutation and copy number alteration (CNA) frequencies of High cluster with those of Low cluster. The driver genes including *PTK2B*, *ATM*, *PIK3C2B*, and *KIT* were more frequently mutated in samples of Low cluster (Figure 2C), suggesting that these driver genes and their regulating pathways might be associated with the reduced T cell infiltration. In addition, more frequent gains in 1q21.3, 2p24.1, 5p15.33, 7q21.2, 7q31.2, 9q34.3, 13q34, and 17q22, and deletions in 2q37.3, 6q27, and 17p13.1, were observed in samples of Low cluster (Figure 2D). Specifically, CNAs of *TERT* located within 5p15.33 and *HDAC4* within 2q37.3 were found to significantly affect their RNA expressions (Supplementary Figure S2). Collectively, somatic mutations and CNAs might be associated with T cell infiltrating levels in HCC.

### Cell Cycle Progression Is Associated With the Reduction of T Cell Infiltration

**FIGURE 3:**
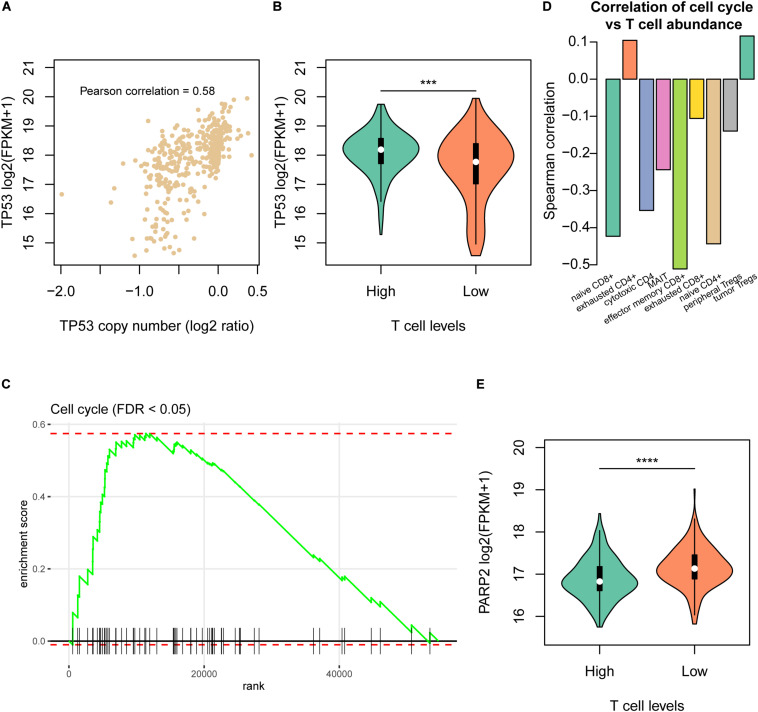
The molecular signatures of the Low T cell level cluster. **(A)** The correlation between TP53 copy numbers and mRNA expression levels. The x-axis and y-axis represent the log2 (copy number/2) and log2 (FPKM + 1). **(B)** The TP53 mRNA expression levels and statistical significance in the High and Low T cell level clusters. **(C)** The enrichment level of cell cycle pathway by the differentially expressed genes between the two T cell level clusters. **(D)** The correlation coefficients between T cell abundances and cell cycle activity in the validation dataset (SRP068976). **(E)** The PARP2 mRNA expression levels and statistical significance in the High and Low T cell level clusters. ***p < 0.001; ****p < 0.0001.

As the CNAs more frequently occurred in samples with low T cell infiltration, we then investigated the driver genes located within those CNAs and the downstream signaling pathways associated with the T cell infiltration. Remarkably, copy numbers of *TP53* were highly correlated with its expression in HCC (Figure 3A), and TP53 expression was downregulated in samples of Low cluster (Figure 3B). Moreover, cell cycle progression was also significantly enriched by the upregulated genes in samples of Low group (Figure 3C), indicating that cell cycle progression was closely associated with reduced T cell infiltration. Moreover, we also validated this finding in the independent dataset and found that cell cycle activity had significant negative correlation with the abundances of naive CD8^+^, cytotoxic CD4, effector memory CD8^+^, MAIT, and naive CD4^+^ (Figure 3D). Furthermore, we also investigated the potential drugs for the upregulated genes in samples of Low group. Notably, PARP2, one of the poly (ADP-ribose) polymerases, was upregulated in samples with low T cell infiltration (Figure 3E), giving us a hint that PARP inhibitors like Olaparib might be used as one of the potential drugs for Low cluster HCC.

### The Immune Checkpoint Regulators Contribute to HCC Immune Evasion

**FIGURE 4:**
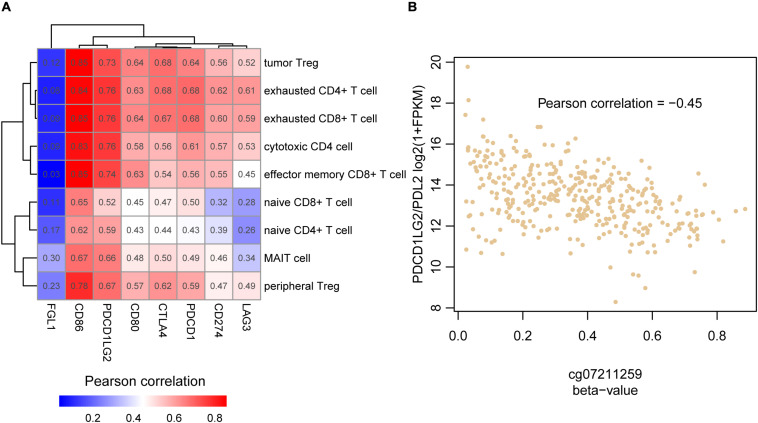
The association between the T cell infiltrating levels and immune checkpoint genes. **(A)** The correlation coefficient matrix between the HCC-infiltrating T cells and immune checkpoint genes. **(B)** The reverse correlation between PDCD1LG2 promoter (cg07211259) methylation and mRNA expression.

As the High cluster was highly infiltrated by the T cell subpopulations, we wondered whether the immune evasion in HCC was regulated by immune checkpoint proteins. The correlation analysis was then conducted to associate the immune checkpoint receptors and their corresponding ligands including PDCD1 (PD-1), CD274 (PD-L1), PDCD1LG2 (PD-L2), CTLA4, CD86, CD80, LAG3, and FGL1 and the nine types of T cells. All combinations of immune checkpoint gene and T cell pairs showed positive correlation (Figure 4A), indicating that the immune checkpoint inhibitors might be used as therapeutic candidates for High cluster samples. Particularly, correlations of *CD86* and *PDCD1LG2* were dominantly higher than those of other immune checkpoint genes. In general, the ligands could be secreted from the tumor cells, and their RNA expression levels might be regulated at genomic or epigenetic levels. Consistently, *PDCD1LG2* was negatively correlated with its promoter methylation level (Figure 4B, Pearson correlation = −0.45), indicating that promoter hypomethylation of *PDCD1LG2* in HCC might be one of the causes of its upregulation and immune evasion.

### The Clinical Significance of the T Cell Infiltrating Levels in HCC

**FIGURE 5:**
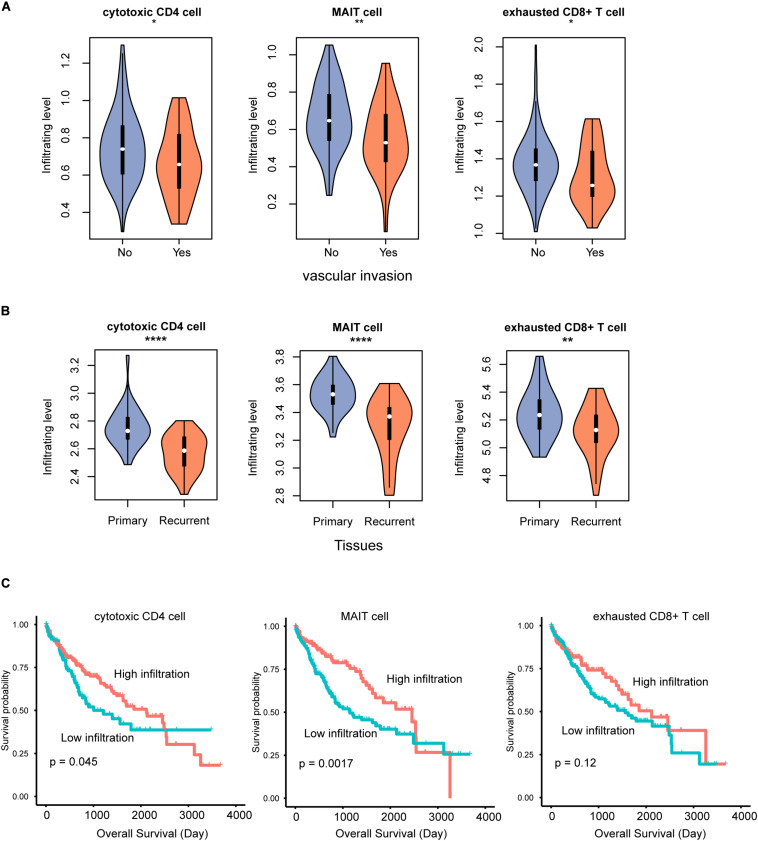
The T cell subpopulations associated with HCC prognosis. The infiltrating levels of cytotoxic CD4, MAIT, and exhausted CD8 + T cells are reduced in HCC with vascular invasion **(A)** and recurrence **(B)**. The association between the infiltrating levels of three T cells and HCC overall survival **(C)**. *p < 0.05; **p < 0.01; ****p < 0.0001.

To further explore the potential clinical significance of the T cell infiltrating levels in HCC, we collected another two gene expression datasets and estimated the T cell infiltrating levels for those samples. The relative infiltrating levels of cytotoxic CD4, MAIT, and exhausted CD8^+^ cells were reduced in samples with vascular invasion (GEO number: GSE20017, Figure 5A, *P* < 0.05) and recurrent HCC tissues (GEO number: GSE56545, Figure 5B, *P* < 0.05), respectively, indicating that the lower infiltrating level might cause worse prognosis. Furthermore, we also tested the prognostic values of the three cell types and observed that high infiltrating levels of cytotoxic CD4 and MAIT cells might be favorable indicators for HCC overall survival (Figure 5C, *P* < 0.05).

## Discussion

The tumor-infiltrating T cells in tumor immunity are now widely accepted to act as the vital roles in HCC. However, due to the different functionality and clinical significance, a further illustration of HCC-infiltrating T cells is needed. In this study, we estimated the HCC-infiltrating levels of T cells using gene expression data and marker genes of T cells. Specifically, the infiltrating levels of all the T cells were significantly reduced in HCC as compared with non-tumor tissues. Accordingly, the marker genes were downregulated in tumor tissues. Particularly, tumor Tregs. and exhausted CD8^+^ T cells were shown to have higher infiltrating levels in HCC tissues. Tregs. are well-recognized to suppress anti-tumor immune response (Curiel, 2008), and high infiltration level of Tregs in HCC is associated with poor prognosis (Lin et al., 2013). The CD8 T cells are usually exhausted in cancer with decreased effector function and proliferative capacity, partly caused by overexpression of inhibitory receptors, such as programmed cell death (PD-1) (Hashimoto et al., 2018). The HCC samples could be clearly classified into two groups by the tumor-infiltrating levels of the T cells. The driver genes including *PTK2B*, *ATM*, *PIK3C2B*, and *KIT* and several CNAs were observed to be associated with reduced T cell infiltrating levels. Moreover, these mutations were located within vital kinases regulating multiple intracellular transduction signaling pathways, such as chemokine signaling pathway, cell cycle, and PI3K-Akt signaling (Hosgood et al., 2008; Todd et al., 2014; Dios-Esponera et al., 2015; Russo et al., 2015). Particularly, deletion of *TP53* more frequently occurred in low T cell infiltration HCC samples and resulted in its downregulation and cell cycle progression. The mutations in *ATM*, deletions in *TP53*, and cell cycle progression indicated that cell cycle progression was closely associated with reduced T cell infiltration. The co-occurrence of cell cycle progression and low immune cell infiltration has been found in osteosarcoma (Scott et al., 2018). In contrast, for the samples with high infiltration T cells, its immune evasion might be regulated by the immune checkpoint regulators, such as PD-1/PD-L1 and CTLA4.

Furthermore, analysis of differentially expressed genes between the two groups revealed that *PARP2* and immune checkpoint regulators, such as PD-1, PD-L1, PD-L2, and *CTLA4* were specifically upregulated in the Low and High groups, respectively. To discover the drug targets for the Low clusters, we found that PARP2 was highly upregulated and DNA repair damage was more frequently observed in Low cluster. Taken together, Olaparib, one of the PARP inhibitors, and immune checkpoint inhibitors might be therapeutic candidates for the samples from the two T cell infiltrating clusters, respectively. Olaparib has been found to have synergistic inhibition of hepatocellular carcinoma growth with suberoylanilide hydroxamic acid (SAHA) (Zhang et al., 2012). Recently, Camrelizumab, one PD-1 inhibitor, showed antitumor activity in pretreated Chinese patients with advanced hepatocellular carcinoma in a multicenter, open-label, parallel-group, randomized, phase-2 trial (Qin et al., 2020).

Clinically, the tumor-infiltrating levels of cytotoxic CD4 cell, mucosal associated invariant T (MAIT) cell, and exhausted CD8^+^ T cell were found to be reduced in not only primary HCC tissues with vascular invasion but also in recurrent HCC tissues, suggesting that reduced infiltrating levels of these T cells might be an indicator of poor prognosis. Consistently, the samples with higher levels of cytotoxic CD4 and MAIT cells had longer overall survival than others, which was consistent with previous studies (Gao et al., 2007; Zheng et al., 2017).

In summary, we systematically evaluated the clinical significance and potential molecular mechanism of tumor-infiltrating T cell subpopulations in hepatocellular carcinoma, which might broaden our insights into the immunological features of HCC.

## Data Availability Statement

All datasets generated for this study are included in the article/Supplementary Material.

## Author Contributions

JJ: guarantor of integrity of the entire study and manuscript review. JL: study concepts and design and definition of intellectual content. JZ: literature research. SK: clinical studies and experimental studies. CW: data acquisition, data analysis, and statistical analysis. DW: manuscript preparation and manuscript editing. All authors contributed to the article and approved the submitted version.

## Conflict of Interest

The authors declare that the research was conducted in the absence of any commercial or financial relationships that could be construed as a potential conflict of interest.

## Supplementary Material

Supplementary Figure 1 The relative abundance of nine T cell subpopulations in non-tumor and HCC in SRP068976. The orange and blue violins represent the HCC and non-tumor tissues.

Supplementary Figure 2 Correlation analysis of the RNA expression levels of TERT or HDAC4 and their copy number ratios.
