# Supplementary figures and images for: Functional and Clinical Characterization of Tumor-Infiltrating T Cell Subpopulations in Hepatocellular Carcinoma
Source: Front Genet. 2020 Sep 30;11:586415. doi: 10.3389/fgene.2020.586415 (PMC7561438; doi:10.3389/fgene.2020.586415)

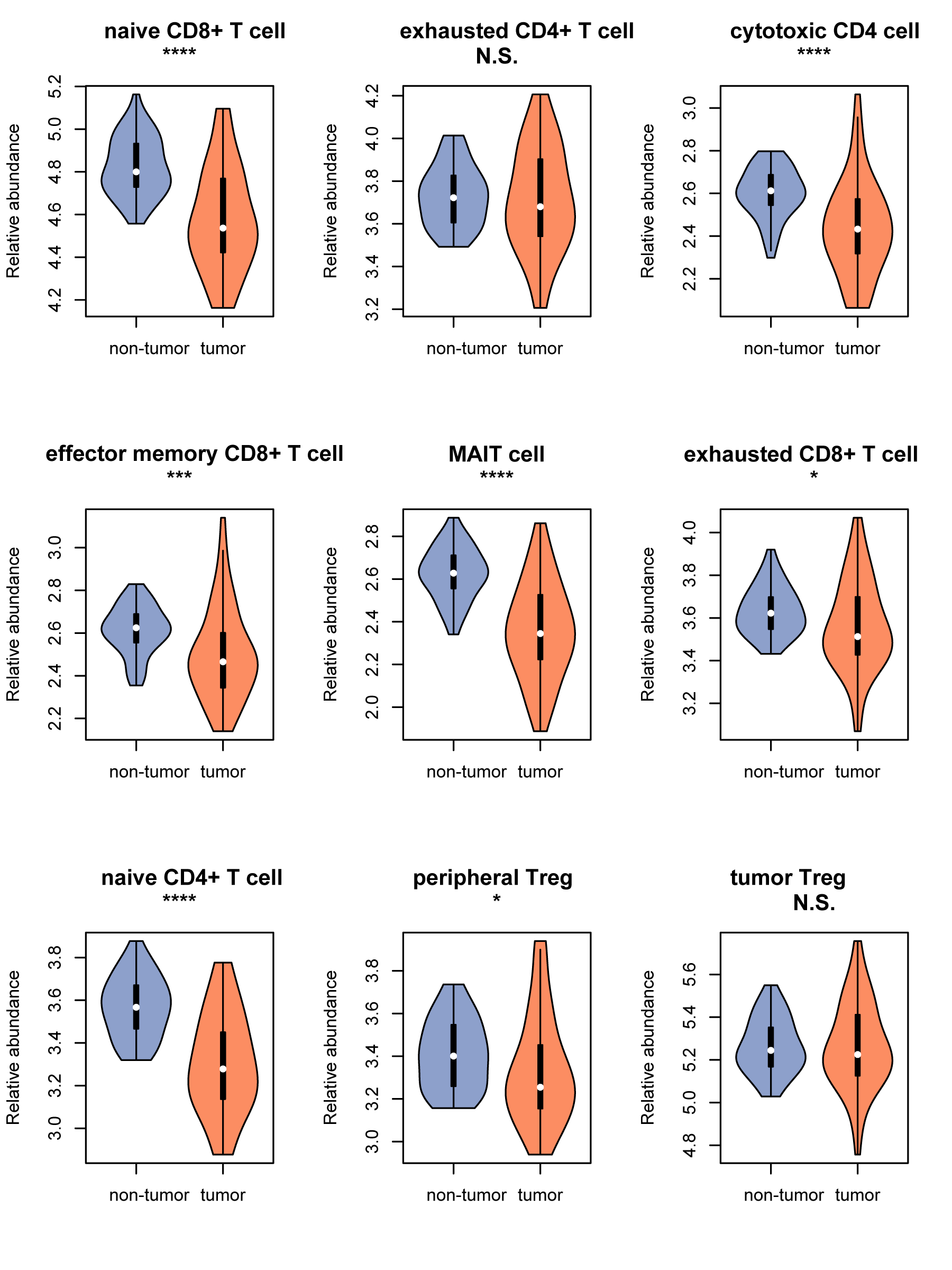

Supplement: Supplementary Figure 1 — The relative abundance of nine T cell subpopulations in non-tumor and HCC in SRP068976. The orange and blue violins represent the HCC and non-tumor tissues. [file Image_1.TIF]

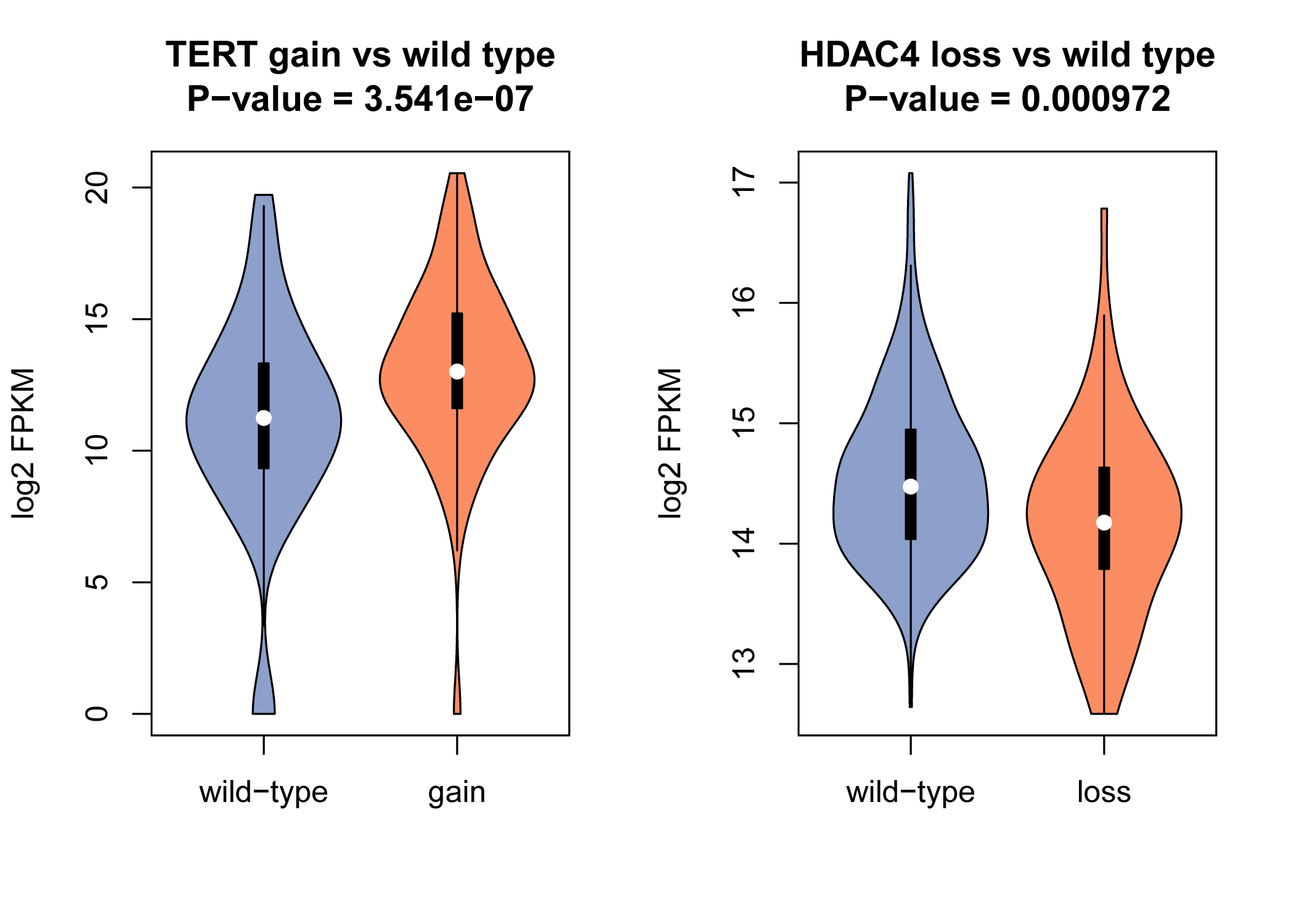

Supplement: Supplementary Figure 2 — Correlation analysis of the RNA expression levels of TERT or HDAC4 and their copy number ratios. [file Image_2.TIF]
